# Stress will kill you anyway!

**DOI:** 10.1038/s41419-020-2433-0

**Published:** 2020-04-06

**Authors:** Alexia Belavgeni, Stefan R. Bornstein, Andreas Linkermann

**Affiliations:** 1 Division of Nephrology, University Hospital Carl Gustav Carus at the Technische Universität Dresden, 01307 Dresden, Germany; 2 Department of Internal Medicine III, University Hospital Carl Gustav Carus at the Technische Universität Dresden, 01307 Dresden, Germany; 3 Biotechnology Center, Technische Universität Dresden, 01307 Dresden, Germany

**Keywords:** Apoptosis, Adrenal gland diseases

## Abstract

Adrenocortical carcinomas are devastating cancers, expressing high levels of glutathione peroxidase 4 (GPX4). Therefore, it was recently suggested that these tumors might be therapeutically targeted by inducing ferroptosis. In this issue of *Cell Death and Disease*, Weigand et al. describe the organ-specific production of steroid hormones as a potential culprit for the exquisite sensitivity of these cancers to ferroptosis.

Ferroptosis, like all necrotic-type cell death pathways, causes an immune reaction referred to as necroinflammation^1^. In the heart, ferroptosis results in rapid neutrophil infiltration^2^, a response that would ideally be directed against cancers. This is of particular importance when current strategies hardly provide tumor regression, and cancers are treated with nonspecific drugs, which induce massive side effects. Adrenocortical carcinomas (ACCs) are just like that. The oncologist’s despair may best be exemplified by common prescription of mitotane, a nonspecific chemotherapeutic derived from the pesticide DDT (1,1-(dichlorobiphenyl)-2,2-dichloroethane) with unknown mechanisms of action.

The association of hormone production and the increase of reactive oxygen species (ROS) in the adrenal gland lead to the hypothesis of a tightly balanced redox system. Given the current knowledge on ferroptosis, Weigand et al.^3^ questioned how this endocrine system might evade cell death upon tumorigenesis. More specifically, with the help of a public database, the Human Proteome Map, it was demonstrated that the adrenal gland expresses a variety of genes, linked to ferroptosis, including glutathione peroxidase (GPX4), lysophosphatidylcholine acyltransferase 3 (LPCAT3), and Acyl-CoA synthase long-chain family member 4 (ACSL4). The expression of GPX4 and ACSL4, two proteins that are centrally involved in the regulation of ferroptosis, was confirmed in human normal adrenal glands (nAGs), adrenocortical adenomas (ACAs), and adrenocortical carcinomas (ACCs). ACCs showed a significantly higher expression of GXP4 mRNA expression compared with nAGs, further underlining the susceptibility of the adrenal gland to ferroptosis. Peroxidation of high levels of arachidonic acid and adrenic acid, both of which are long-chain polyunsaturated fatty acids (PUFAs), was detected in the cortex of nAGs, ACA, and ACC samples.

Cell culture systems are frequently used to study the adrenal gland and its malignancies. A well-known and established human ACC cell line is NCI-H295R that was employed in this study alongside with the less frequently investigated CU-ACC1 and CU-ACC2 cell lines. By western blot analysis, all of these cell lines were demonstrated to express significant amounts of ACSL4 and GPX4. The above-mentioned findings were in keeping with the concept of the adrenal gland to be sensitive toward ferroptosis.

Induction of ferroptosis is routinely performed by stimulation with several small molecules that may be classified as four categories of ferroptosis inducers (FINs). The two most commonly used FINs are type I FINs, e.g., erastin (inhibitors of system X^c-^) and type II FINs, such as RSL3, that directly inhibit GPX4. Weigand et al. challenged all three ACC cell lines with different concentrations of erastin, but failed to induce significant amounts of ferroptosis. However, NCI-H295R, CU-ACC1, and CU-ACC2 were highly sensitive to RSL3 treatment, while non-steroidogenic cell lines were less sensitive to RSL3 treatment.

Hormone production is one of the hallmarks of the adrenal gland. Testing the hypothesis of a potential connection between the hormone production pathways and ferroptosis (Fig. 1), Weigand et al. challenged the ACC cell lines with RSL3 in the presence of the pan-steroid inhibitor ketoconazole. As expected, ketoconazole impaired hormone production. Remarkably, at concentrations of 25 μΜ, it significantly inhibited RSL3-induced cell death of all ACC cell lines.

**Fig. 1:**
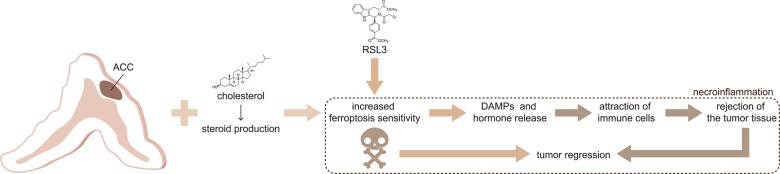
Steroid-producing adrenocortical carcinomas are sensitive to ferroptosis induction. Steroid production in the adrenal gland is associated with high levels of intracellular reactive oxygen species that originate from the respiratory chain and may trigger Fenton reactions and finally cause lipid peroxidation. Glutathione peroxidase 4 (GPX4), a master regulator of ferroptosis, inhibits lipid peroxidation in such cells. During tumorigenesis, steroid-producing adrenocortical carcinomas (ACCs) may become partially dependent on GPX4 expression. A targeted therapy with RSL3, a compound that covalently binds to the active center of GPX4, results in dysfunction of the anti-ferroptosis protein and thereby kills the cancer by ferroptosis. DAMP release from the ferroptotic tumor, in addition, might potentiate the antitumor effect by recruiting neutrophils and other immune cells to reject the tumor tissue.

Steroid synthesis involves several enzymes, including 11-β-hydroxylase that is responsible for the generation of cortisol and corticosterone from 11-deoxycortisol and deoxycorticosterone, respectively. A more specific inhibitor of 11-β-hydroxylase, metyrapone, did not affect the sensitivity to RSL3-mediated ferroptosis in various concentrations investigated.

As discussed above, the mechanism of action of mitotane remains elusive. The authors report that increased concentrations of mitotane-induced cell death of the NCI-H295R cells showed a concentration-dependent increase in lipid peroxidation. This interesting finding resulted in the hypothesis of a ferroptotic type of cell death induced by mitotane. Therefore, liproxstatin-1, a ferroptosis inhibitor, was used in different concentrations in parallel with different concentrations of mitotane, and all ACC cell lines were treated. Nevertheless, none of the investigated combinations reversed mitotane-induced cell death. Given the high sensitivity of the ACC cell lines to RSL3, the authors of this study investigated potential synergistic effects of mitotane and RSL3 treatment. Significant changes of cell death induction were observed only in the co-treatment of NCI-H295R and CU-ACC2 cell lines with mitotane and RSL3, compared with monotherapy.

In summary, two recent publications, one published in *Cell Death and Disease*^3^, and the other in the *Proceedings of the National Academy of Sciences* (PNAS)^4^, describe ACC sensitivity to ferroptosis induction. Both groups consistently found high protein expression levels of GPX4 in those cells. GPX4 was known to encompass anti-ferroptotic properties, and it was therefore expected that these tumors are sensitive to ferroptosis induction by the GPX4 inhibitor RSL3^5^. Besides GPX4, however, other breaks on ferroptosis have recently been reported. Most prominently, the discovery of FSP1 (ferroptosis-sensitizing protein 1, previously known as apoptosis-inhibiting factor m2 (*AIFM2*)) as a GSH-independent inhibitor of ferroptosis^6,7^, will give rise to further investigation in ACCs. Exploration of the sensitivity of ACCs toward other types of FINs will follow soon. Finally, both groups reported the inability of ferroptosis inhibitors to block the mitotane-induced cell death. These findings indicate another type of necrotic, potentially toxic, cell death.

In conclusion, the discovery of ACCs as ferroptosis-sensitive tumors will hopefully trigger further investigation in primary tumor samples. It is, however, not trivial to detect ferroptosis in human tissues^8^. Nonetheless, we strongly support to test ferroptosis inducers as *ultima ratio* for patients with metastasized ACCs as soon as they are available for clinical application.
